# Explainable cluster-based learning for prediction of postprandial glycemic events and insulin dose optimization in type 1 diabetes

**DOI:** 10.1371/journal.pdig.0000996

**Published:** 2025-09-16

**Authors:** Najib Ur Rehman, Ivan Contreras, Aleix Beneyto, Josep Vehi

**Affiliations:** 1 Department of Electrical, Electronic and Automatic Engineering, Institut d’Informatica i Applicacions, Universitat de Girona, Girona, Spain; 2 Professor Serra Hunter, Universitat de Girona, Girona, Spain; 3 Centro de Investigacion Biomedica en Red de Diabetes y Enfermedades Metabolicas Asociadas (CIBERDEM), Instituto de Salud Carlos III, Madrid, Spain; Fundação Oswaldo Cruz: Fundacao Oswaldo Cruz, BRAZIL

## Abstract

Effective management of postprandial glycemic excursions in type 1 diabetes requires accurate prediction of adverse events and personalized insulin adjustments informed by interpretable models. This study presents an explainable dual-prediction framework that simultaneously forecasts postprandial hypoglycemia and hyperglycemia within a 4-hour window using cluster-personalized ensemble models. Glycemic profiles were identified through a hybrid unsupervised approach combining self-organizing maps and k-means clustering, enabling the training of specialized random forest classifiers. The system outperformed baseline models on both real-world and simulated datasets, achieving high performance (AUC = 0.84 and 0.93; MCC = 0.47 and 0.73 for hypo- and hyperglycemia, respectively). Model interpretability was addressed using global (SHAP) and local (LIME) explanations, while interaction analysis revealed the non-linear effects of carbohydrate intake and insulin bolus combinations. An insulin adjustment module further refined pre-meal bolus recommendations based on predicted risk. Simulated evaluations confirmed improved postprandial time-in-range and reduced hypoglycemia without excessive hyperglycemia. These results underscore the potential of profile-driven and explainable machine learning approaches to support safer, individualized diabetes care.

## Author summary

Managing blood glucose after meals is a major challenge for subjects with type 1 diabetes, as both high and low glucose levels can occur depending on meal content, pre-meal insulin dosage, and individual variability. Predicting these postprandial events early can help prevent adverse episodes. Many advanced prediction systems act as black boxes, making them difficult for patients and healthcare providers to trust. In this study, we developed an explainable machine learning framework that predicts both hypoglycemia and hyperglycemia events within four hours after a meal. Our method first groups meal events into similar glycemic profiles using clustering, then trains specialized models for each profile to improve accuracy. We also use two methods, SHAP and LIME, to explain how the models make their predictions. Finally, we proposed a method to adjust pre-meal insulin doses based on predicted events. Tests with real patients and simulated data showed that our approach improves prediction accuracy, reduces post-meal hypoglycemia, and maintains safe glucose ranges. By combining personalization with interpretability, this work offers a practical step toward safer, more adaptive insulin management in everyday life.

## 1. Introduction

Type 1 diabetes (T1D), often diagnosed in childhood or adolescence, is a chronic autoimmune condition in which the body destroys the insulin-producing *β*-cells in the pancreas, resulting in insufficient insulin production. This leads to hyperglycemia, or elevated blood glucose (BG) levels, which, if not controlled, can result in long-term complications affecting vital organs such as the heart, kidneys, eyes, and nerves [1–3]. In severe cases, hyperglycemia can also progress to diabetic ketoacidosis [4–6]. Hyperglycemia can be categorized as level 1 (BG: 180—250 mg/dL) or level 2 (BG above 250 mg/dL) [7]. Additionally, T1D subjects are at risk of hypoglycemia, characterized by low BG levels, which may result from excessive insulin administration, missed meals, or intense physical activity and can lead to severe outcomes such as seizures or loss of consciousness if untreated [8–10]. Hypoglycemia can also be classified as level 1 (BG: 54—70 mg/dL) or level 2 (BG below 54 mg/dL) [8]. Following meal intake, individuals with T1D often experience substantial changes in BG levels, commonly referred to as postprandial BG excursions. A BG excursion means the rise and subsequent fall in glucose concentration that occurs after a meal. These excursions are influenced by various factors, including the quantity and composition of the meal, insulin administration, and individual metabolic variability. These fluctuations can be particularly challenging to manage and are associated with an increased risk of both acute and long-term complications. Therefore, effective management of BG excursions, especially in the postprandial period, is essential for individuals with T1D to reduce the risk of adverse health outcomes [11].

Understanding and managing factors such as food intake, physical activity, stress, illness, and insulin effects is key to improving outcomes for T1D subjects [12]. However, individual responses to these factors vary widely, motivating the need for glycemic profiling approaches that identify meaningful subgroups based on observed glucose behavior. Severe glycemic events, especially those reaching level 2 severity, pose immediate health risks and increase the potential for long-term complications [13]. Accurately predicting and managing these events is crucial for maintaining glycemic control, improving quality of life, and reducing health risks [14]. Continuous glucose monitoring (CGM) is a transformative tool in this effort, providing real-time glucose estimates that help users manage hypoglycemia and hyperglycemia, balance daily glucose fluctuations, and improve long-term HbA1c levels, particularly for those with poor BG control [15,16].

It has become essential for individuals using multiple daily injections (MDI) for insulin delivery, assisting in adjusting insulin doses based on real-time glucose trends [17]. However, challenges remain, especially in the postprandial period, where BG can fluctuate significantly due to meal-derived glucose absorption [18,19].

To improve BG control during the postprandial window, modern CGM devices offer real-time alerts based on pre-set thresholds. However, there is an increasing emphasis on the potential of predictive alerts [19]. Classical algorithms have been developed to predict hypoglycemia with a short lead time [20–22]. However, the integration of more sophisticated statistical and machine learning (ML) models offers the promise of enhanced prediction accuracy and a longer predictive horizon [23]. Even though advanced ML models have shown promising results in predicting glycemic events, their adoption in clinical practice hinges on their explainability [20,21]. Healthcare providers and patients require transparent, interpretable models to understand the rationale behind predictions and make informed decisions [24]. This is particularly critical for T1D management, where incorrect insulin adjustments based on unclear predictions can lead to severe health consequences. Therefore, integrating explainable AI ensures trust and usability while maintaining robust performance metrics [25].

Furthermore, there is a critical need to integrate predictive models with bolus calculators to provide actionable insulin dosing recommendations based on anticipated glycemic trends. Currently, bolus calculators primarily rely on deterministic algorithms and manual input of carbohydrate intake and BG levels; incorporating predictive alerts based on CGM data could enhance their functionality. However, despite the potential, research on leveraging these predictive models to feed bolus calculators remains sparse. This gap underscores the need for developing systems that not only predict glycemic trends but also seamlessly integrate these predictions into actionable insulin dosing recommendations [26–28].

The aim of this study was to devise a system that excels in predicting postprandial glycemic events while prioritizing user-centric design, reducing patient data input burden and optimizing health outcomes [29,30]. In light of the aforementioned challenges, the following contributions are made:

- A dual-prediction system that simultaneously forecasts both hypoglycemic and hyperglycemic events within a 4-hour postprandial period.
- An integrated learning approach that performs data-driven glycemic profiling and leverages it through personalized ensemble models to enhance predictive performance and adaptability.
- An interpretable framework that integrates SHAP- and LIME-based explanations to foster transparency and clinical trust.
- Meal bolus optimization based on data-driven predictions to dynamically adjust insulin dosing and improve postprandial glycemic control.

## 2. Methodology

### 2.1. Experimental datasets and preprocessing

Data from the REPLACE-BG study, comprising people diagnosed with T1D, was used to validate our approach [31]. The dataset contains CGM data from 227 adults with a mean age of 44 ± 14 years and an average diabetes duration of 24 ± 12 years. All participants utilized insulin pumps during a 26-week clinical trial conducted in free-living conditions. Data from participants with over 50% missing data or lacking meal-related information were excluded, resulting in 179 participants being eligible for this study. For each participant, CGM data along with timestamps, estimated meal carbohydrates, and bolus insulin dosages were extracted and utilized for the study. Table 1 presents the time-in-range percentages and associated statistical metrics for the selected participants.

**Table 1:** Summary of CGM data metrics for real patients.

Metrics	Real Patients
% Time below 54 mg/dL	0.5 [0.29 - 1.20]
% Time between 54-70 mg/dL	2.1 [1.40 - 3.54]
% Time between 70-180 mg/dL	61.0 [55.73 - 69.92]
% Time above 180 mg/dL	22.8 [21.01 - 27.97]
% Time above 250 mg/dL	7.3 [5.48 - 12.10]
Median BG (mg/dL)	152.4 [142.50 - 163.50]
Mean BG (mg/dL)	156.0 [148.73 - 170.53]
Coefficient of Variation (%)	37.0 [34.66 - 40.37]
Standard Deviation (mg/dL)	59.13 [52.65 - 66.53]

A second virtually generated dataset, created using a customized simulator based on the Dalla Man model [32], was used to test the proposed ML model in an in silico environment with a bolus adjustment algorithm to validate the model performance in simulation time. A total of 10 adult patients were considered for a simulation spanning 120 days for each patient, with three meals provided per day. Additional variability was added to the dataset generation scenarios, such as meal-time variation (± 20 minutes) and meal content (± 20%). To include intrapatient variability, circadian variations were incorporated into the insulin sensitivity and meal absorption rate for each patient during the simulation [33]. Furthermore, carbohydrate misestimation was integrated with a normal distribution (± 40%) to mimic real-life scenarios. The datasets used in this study are provided as Supplementary Information (S1 Dataset).

Table 2 provides a comprehensive summary of the mean values for various time-in-range percentages and statistical metrics of the simulated patient dataset.

**Table 2:** Summary of CGM data metrics for simulated patients.

Metrics	Simulated Patients
% Time below 54 mg/dL	4.5[2.22 - 7.27]
% Time between 54-70 mg/dL	4.2[3.02 - 5.47]
% Time between 70-180 mg/dL	78.1[74.97 - 84.33]
% Time above 180 mg/dL	6.8[5.83 - 11.50]
% Time above 250 mg/dL	1.0[0.62 - 2.41]
Median BG (mg/dL)	119.4[116.72 - 126.71]
Mean BG (mg/dL)	122.2[121.07 - 132.45]
Coefficient of Variation (%)	36.0[31.97 - 38.84]
Standard Deviation (mg/dL)	43.25[39.33 - 47.15]

### 2.2. Feature engineering

In this study, 13 time-domain features were extracted from CGM data, meal information, and meal insulin bolus (MIB). These were selected from an original set of 27 candidates evaluated in our prior study [34]. In the previous study, we used ANOVA F-measure rankings to identify the most informative. Features with the lowest scores were excluded from the feature set, such as area under the curve, insulin on board, and rate of change for this study. Additionally, we use an input feature based on the clustering methodology described in Sect 2.3. The resulting subset captures temporal dynamics relevant to postprandial glycemic responses while maintaining model simplicity. These included:

- **CGM at meal**: BG value observed at the start of meal (*m*_0_).
- **Mean CGM meal**: average glucose values for each of the six hours preceding the meal, i.e., mean(BG(*m*_0_-1), BG(*m*_0_-2),..., BG(*m*_0_-6)).
- **Low and high blood glucose index (LBGI and HBGI)**: calculated over the 6 hours prior to the meal to quantify the risk of hypo- and hyperglycemia, respectively.
- **Glucose excursions**: the difference between the BG value at meal time and 60 minutes before.
- **Estimated carb intake (CHO)**: meal-related carbohydrate amount (grams), as recorded or estimated.
- **Meal time (hour)**: time-of-day indicator (discrete hour) when the meal is consumed.
- **Meal insulin bolus (MIB)**: insulin dose administered at meal onset (units).
- **Cluster label**: a numeric identifier assigned after unsupervised clustering, indicating glycemic subgroup membership.

**Fig 1:**
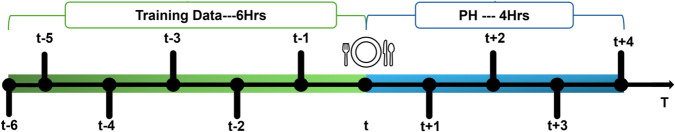
Illustration of pre- and postprandial time windows. Where t is the hour of the mealtime and PH: prediction horizon.

Postprandial hypoglycemia and hyperglycemia were evaluated over a 4-hour prediction horizon following meal onset (defined by the timestamp of carbohydrate ingestion).In real-world data, meal times were self-reported and naturally distributed throughout the day. In contrast, the in silico dataset used three standardized meals per day (breakfast, lunch, dinner) with randomized timing offsets of ±20 minutes to simulate routine variability. Fig 1 illustrates the full timeline used per instance—including the 6-hour retrospective window used to extract input features and the 4-hour postprandial horizon for outcome labeling. We define two hyperglycemic detection thresholds during a postprandial period, with the first two hours tracking levels ≥ 250 mg/dL and the next two hours tracking levels ≥180 mg/dL. In the first two hours, higher metabolic activity justifies a hyperglycemia threshold of ≥250 mg/dL. As metabolic processes stabilize in the next two hours, a lower threshold of ≥180 mg/dL identifies persistent hyperglycemia, offering detailed glucose metabolism characterization. Hypoglycemia event during the postprandial window was identified if BG measurements are ≤70 mg/dL and sustained for at least 15 minutes within the monitoring window. Binary indicators (1 for abnormal event, 0 for no event) classify these occurrences. To improve clarity, we adopt the following notations for the two binary classification systems throughout the article: S1 for hypoglycemia prediction and S2 for hyperglycemia prediction.

### 2.3. Glycemic profiling via clustering

To address inter-individual variability in glycemic behavior and enhance predictive performance, we implemented a two-stage unsupervised clustering pipeline following feature extraction. This approach served as a data-driven glycemic profiling strategy, aimed at identifying behavioral subgroups among meal instances with similar pre-meal glycemic profiles. Firstly, the original training datasets were first divided into four subsets based on event labels: hypoglycemia, non-hypoglycemia, hyperglycemia, and non-hyperglycemia. Each instance was encoded as a 12-dimensional feature vector, excluding the cluster label

Within each subset, a Self-Organizing Map (SOM) [35] with a 10×10 grid was independently trained to preserve topological similarity and represent pre-meal patterns through localized neuron weights. Once the SOMs had converged, the resulting weight vectors, which captured representative profiles of pre-meal glycemic behavior, were passed to the next stage for cluster assignment.

To reduce complexity and organize these prototypes into meaningful categories, k-means clustering was applied to the SOM weight vectors. The optimal number of clusters (k = 3) was selected based on the Elbow method and the silhouette score. This yielded three clusters per subset, for a total of twelve behavioral subgroups: three for each of the four glycemic event types.

Each training instance was assigned a cluster label by mapping it to the best-matching SOM neuron and then to its corresponding k-means cluster. These cluster labels served a dual function. First, they were used as additional categorical input features during model training. Second, the centroids of each cluster were stored and later used during inference to assign new test instances based on minimum Euclidean distance. This allowed each instance to be matched with its most representative subgroup, ensuring that predictions leveraged the most contextually relevant classifiers.

**Fig 2:**
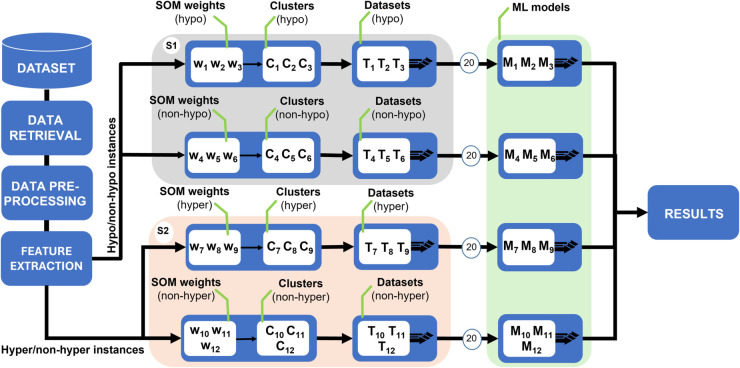
Schematic representation of the proposed methodology, illustrating the steps from data preprocessing to feature extraction, clustering, and model evaluation.

As shown in Fig 2, this clustering mechanism enabled the model to capture glycemic heterogeneity more effectively. It provided a foundation for training specialized classifiers per behavioral subgroup, reducing overgeneralization and enhancing personalization without relying on subject-level identifiers.

### 2.4. Machine learning model

The ML model training was based on a dual-system architecture to handle the two glycemic excursion types separately, with S1 handling hypoglycemia and S2 handling hyperglycemia. Each system comprises six Random Forest (RF) models, trained on distinct profile subgroups identified during the clustering phase (Sect 2.3).

In S1, three models (M_1_, M_2_, and M_3_) were trained specifically on data related to hypoglycemic events, while the other three models (M_4_, M_5_, and M_6_) focused on non-hypoglycemic instances. Similarly, S2 included three models (M_7_, M_8_, and M_9_) specialized in predicting hyperglycemic events, and three others ((M_10_, M_11_, and M_12_) trained on corresponding non-hyperglycemic instances. This setup enabled the system to learn subgroup-specific decision boundaries for both the presence and absence of glycemic excursions, reducing false positives and enhancing generalizability.

Before training, input features were normalized to ensure balanced scale contributions. Each RF model underwent hyperparameter tuning using randomized search with cross-validation. To mitigate bias from a class imbalance in cluster-derived subsets, a 90%/10% train-test split was applied using repeated cross-validation with 20 iterations.

**Fig 3:**
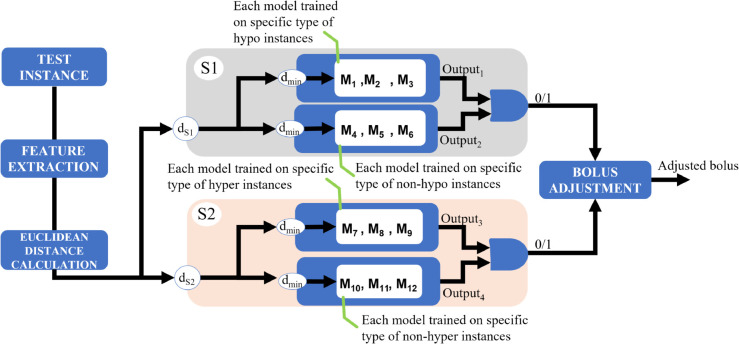
Schematic representation of the test instance classification and bolus adjustment system. Feature extraction and Euclidean distance calculations identify the closest reference models. Systems S1 and S2 predict the respective glycemic events, and the results are combined to guide bolus adjustment.

During inference, each test instance was compared to the stored centroids from the clustering strategy using Euclidean distance [36]. For both systems S1 and S2, the two closest centroids were identified. The RF models associated with those centroids were then selectively activated. This resulted in the evaluation of two specialized models per system. Their binary predictions were combined using a logical AND gate to produce the final classification: 1 (event predicted) only if both models concurred, and 0 otherwise. As shown in Fig 3, this dynamic selection mechanism reduced computational overhead by avoiding the evaluation of all twelve models per instance. More importantly, it ensured that predictions were informed by classifiers trained on highly similar pre-meal glycemic patterns, promoting both efficiency and personalization.

The performance of the ML models was evaluated using several metrics derived from the confusion matrix, including sensitivity, specificity, and accuracy, to provide a detailed assessment. The Matthews correlation coefficient (MCC) was used as the primary metric and also served as the loss function during training due to its robustness in imbalanced classification problems. The receiver operating characteristic (ROC) curves were also plotted to visualize the trade-off between sensitivity and specificity. Data preprocessing and feature engineering were carried out in MATLAB, while clustering, model training, testing, and performance evaluation were implemented using Python (v 3.1) with scikit-learn library.

### 2.5. Explainable artificial intelligence

Explainability was a key design objective to ensure that the system’s predictions are trustworthy, interpretable, and clinically meaningful. To that end, we integrated two complementary techniques: SHAP (Shapley additive explanations) for global model interpretation and feature interaction analysis, and LIME (Local Interpretable Model-agnostic Explanations) for instance-level insights.

SHAP provides insight into how each input feature contributes to the model’s output. It enables researchers and clinicians to identify the most relevant variables influencing binary outcomes and supports personalized interventions such as insulin adjustments or dietary modifications. The SHAP value for a given feature *i* is defined as:

$$\phi_{i}=\sum_{S\subseteq N⧵\{i\}}\frac{|S|!(|N|-|S|-1)!}{|N|!}\left[f(S\cup \{i\})-f(S)\right]$$ (1)

Here, *N* represents the complete set of features (13 time-domain variables) and *S* denotes a subset of them. The function *f*(*S*) indicates the model’s predicted probability of hypoglycemia or hyperglycemia when the prediction is based solely on the features contained within the subset *S*.

SHAP interaction values extend the standard SHAP framework by quantifying how two features jointly drive the model’s output beyond their individual contributions. We applied this analysis specifically to carbohydrate intake (CHO) and insulin bolus (MIB) to reveal their combined influence on postprandial glycemic dynamics. This approach uncovered latent scenarios—such as overdosing or underdosing—where the synergy between CHO and MIB proved more decisive for prediction than either feature alone. For any feature pair *i* and *j*, their combined interaction effect is calculated as:

$$\phi_{i,j}=\sum_{S\subseteq N⧵\{i,j\}}\frac{|S|!(|N|-|S|-2)!}{|N|!}[f(S\cup \{i,j\})-f(S\cup \{i\})-f(S\cup \{j\})+f(S)]$$ (2)

On a local level, LIME was used to explain individual predictions by approximating the complex model with a simple interpretable function around a given data point $x\in {\mathbb{R}}^{13}$. This was achieved by generating perturbed samples $z^{\prime }\in Z$ near *x*, weighting them based on proximity $\pi (x,z^{\prime })$, and solving the optimization:

$$\min_{g\in G}\sum_{z^{\prime }\in Z}\pi (x,z^{\prime }){\left[f(z^{\prime })-g(z^{\prime })\right]}^{2}+\Omega (g)$$ (3)

where $f(z^{\prime })$ is the prediction of the original model for input $z^{\prime }$, $g(z^{\prime })$ is the surrogate interpretable model’s output, and Ω(g) penalizes complexity to favor interpretability. This formulation ensures that *g* approximates the behavior of *f* near *x*, offering an explanation of how the model arrived at its prediction for that specific data point.

### 2.6. Pre-meal insulin adjustment protocols

Building upon the dual-prediction system, we designed a two-stage bolus adjustment algorithm that (i) computes the standard meal bolus, (ii) flags upcoming hypo- or hyperglycemia, and (iii) refines the bolus dose using smooth corrective factors and CGM trends. The insulin adjustment strategy was implemented and tested in a validated in silico simulator based on the Dalla Man glucose–insulin model, which reproduces realistic meal timing, absorption variability, insulin sensitivity fluctuations, and carbohydrate estimation errors.

First, the standard bolus calculator formula is used to calculate the initial MIB as shown in Eq 4. The standard bolus calculator uses factors related to carbohydrate consumption, current glucose levels, and parameters that account for individual variability in insulin sensitivity and glycemic regulation.

$$\text{MIB}=\left(\frac{\text{CHO}}{\text{CR}}\right)+\left(\frac{{\text{CGM}}_{\text{meal}}-{\text{CGM}}_{\text{target}}}{\text{CF}}\right)$$ (4)

where CHO (g) is the estimated amount of carbohydrates consumed during the meal, CR (g/U) is the carbohydrate-to-insulin ratio which determines how many grams of carbohydrates can be effectively managed by one unit of insulin, CF (mg/dL/U) is the correction factor, which defines how much a single unit of insulin will reduce the BG level, ${\text{CGM}}_{\text{meal}}$ (mg/dL) is the BG level at mealtime, and ${\text{CGM}}_{\text{target}}$ (mg/dL) represents the target BG that the system aims to achieve, set to 150 mg/dL in this study.

Once MIB is determined, systems S1 and S2 predict whether the post-meal period will fall outside the safe glycemic range. If no excursion is forecast, MIB is delivered unchanged. Otherwise, we apply a sigmoid-based refinement to obtain a modified bolus (*B*_*mod*_):

$$B_{mod}=MIB\cdot \left(1+adjustment\_factor\right),$$ (5)

$$adjustment\_factor=\sigma ({CGM}_{meal})+\sigma (CF)-1,$$ (6)

$$\sigma (x)=\frac{1}{1+e^{-x}}.$$ (7)

This continuous transform prevents discrete dose perturbations and ensures proportional scaling of the bolus according to both pre-meal glucose deviation and individual insulin sensitivity.

Finally, to account for recent glucose trends, we compute the CGM slope $\Delta_{\text{CGM}}$ over the 60 minutes preceding the meal. If a downward trend in glucose levels is detected alongside either hypoglycemia or hyperglycemia predictions, further adjustments are made based on the trend as shown in Algorithm 1.

Two gain coefficients, *α* and *γ*, were introduced to modulate the initial bolus $B_{initial}$ based on the predicted glycemic event and the CGM trend slope. Determined empirically, α=1.02 applies a 2% uplift to $B_{initial}$ when hypoglycemia is forecast with $\Delta_{CGM}<0$, mitigating excessive dose reduction during falling glucose. Similarly, γ=1.01 imparts a 1% increase to $B_{initial}$ under hyperglycemia risk with a downward trend, ensuring adequate corrective insulin. These empirically derived gains constrain bolus adjustments within clinically safe margins, balancing corrective potency against the risks of over- or under-dosing.

**Algorithm 1: Bolus adjustment algorithm based on glycemic prediction and CGM trend.**

1: **if** S1 predicts hypoglycemia **then**

2:   **if** CGM trend is downward **then**

3:    Further reduce bolus by factor α: $B_{\text{final}}=\frac{B_{\text{mod}}}{\alpha }$ - IOB

4:   **else if** CGM trend is upward **then**

5:    Apply the initial adjusted bolus: $B_{\text{final}}=B_{\text{mod}}$

6:   **end if**

7: **else if** S2 predicts hyperglycemia **then**

8:   **if** CGM trend is downward **then**

9:    Further reduce bolus by factor γ: $B_{\text{final}}=\frac{B_{\text{mod}}}{\gamma }$ - IOB

10:   **else if** CGM trend is upward **then**

11:    Apply the initial adjusted bolus: $B_{\text{final}}=B_{\text{mod}}$

12:   **end if**

13: **end if**

14: **return** $B_{\text{final}}$

## 3. Results

This section presents the performance results of the proposed approach using both real and simulated data. First, we evaluate the predictive capabilities of Systems S1 (hypoglycemia) and S2 (hyperglycemia) on real patient datasets, including an analysis of model interpretability. Second, we perform realistic and challenging in silico simulations to demonstrate the potential of our prediction models in guiding bolus adjustments, with results reported across various time-in-range metrics for both complete and postprandial windows.

### 3.1. Assessment of the proposed approach on real patient data

Table 3 provides a summary of the key metrics for both S1 and S2, with comparisons to baseline models that do not incorporate clustering. Each baseline (Baseline_*hypo*_ and Baseline_*hyper*_) is a binary RF classifier trained to detect hypoglycemia and hyperglycemia, respectively. Optimal decision thresholds were determined by maximizing Youden’s J statistic, and reported metrics correspond to the median performance over 20 iterations.

**Table 3:** Performance metrics for S1 and S2 prediction with comparisons to the baseline models.

Parameter	Hypoglycemia		Hyperglycemia	
	Baseline_*hypo*_	S1	Baseline_*hyper*_	S2
Accuracy (%)	85.10	86.50	62.19	85.60
Mathew Cross-Correlation	0.22	0.47	0.25	0.73
Sensitivity (%)	59.64	72.77	56.21	82.04
Specificity (%)	68.13	77.70	68.79	91.67

**Fig 4:**
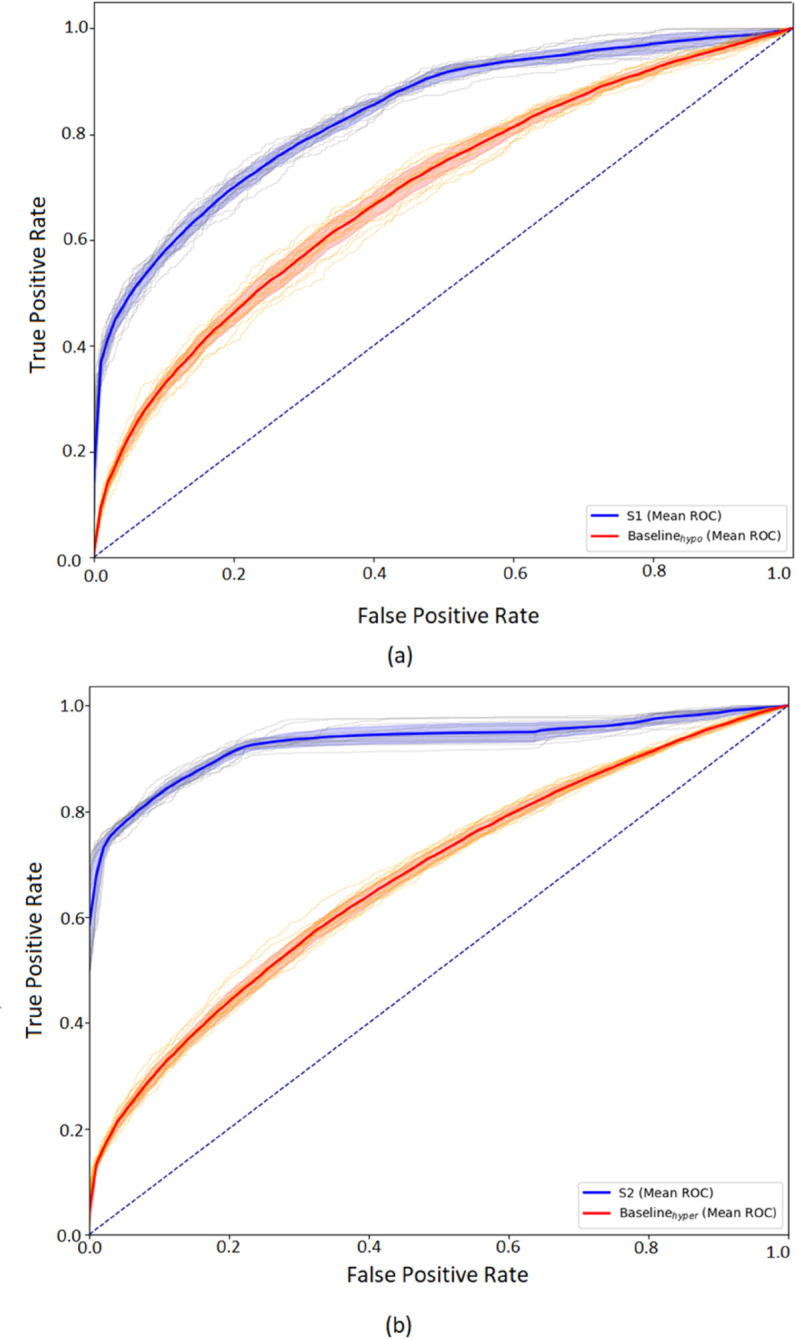
Mean ROC Curves for: (a) Hypoglycemia prediction across all iterations for Baseline_*hypo*_ and S1, and (b) Hyperglycemia prediction across all iterations for Baseline_*hyper*_ and S2.

System S1 showed improved detection of hypoglycemic events, achieving 86.50% accuracy and an MCC of 0.47. Sensitivity and specificity metrics for S1 were 72.77% and 77.70%, respectively. In contrast, the baseline achieved lower scores across all metrics, with an MCC of 0.22. Fig 4a displays the mean ROC curves for hypoglycemia prediction across multiple iterations for both Baseline_*hypo*_ and cluster-based models in System S1. The Baseline_*hypo*_ model achieves an average AUC of 0.69±0.01 while S1 shows improved performance, reaching 0.84±0.01.

System S2 achieved an accuracy of 85.60% and an MCC of 0.73 for hyperglycemia prediction. Sensitivity and specificity values were 82.04% and 91.67%, respectively. Fig 4b presents the mean ROC curves for hyperglycemia prediction across multiple iterations for both the Baseline_*hyper*_ and System S2. The Baseline_*hyper*_ model demonstrates modest predictive capability, with an average AUC value of 0.68±0.01, indicating limited effectiveness in distinguishing hyperglycemic events. In contrast, the cluster-based model significantly improves performance, achieving an average AUC value of 0.93±0.01, showcasing high classification power in predicting hyperglycemia.

### 3.2. Explainable machine learning model

**Fig 5:**
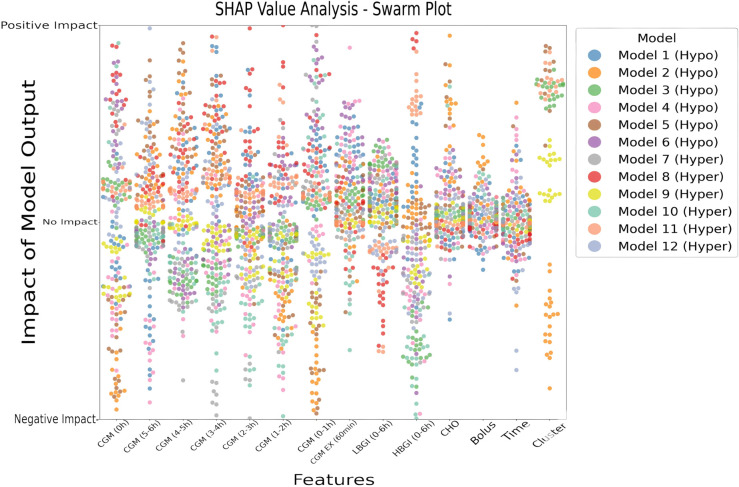
Mean SHAP values for features across all iterations and models. The x-axis represents the features, while each dot shows the mean SHAP value for each iteration against each model, illustrating the magnitude and direction of the feature’s impact on the model’s prediction. The y-axis categorizes the impacts as positive, negative, or neutral for interpretability.

To better understand how different input variables influence predictions in Systems S1 and S2, we analyzed feature attributions using SHAP. Fig 5 offers a detailed visualization of feature contributions across models using SHAP values. Each dot represents a single model within a cluster, and their horizontal spread highlights the range of feature impacts.

For both S1 and S2, recent CGM values (particularly at 0h and 1–2h) exhibit wide distributions along the x-axis, reflecting both strong positive and negative contributions depending on context. Short-term glucose variability (CGM EX (60min)) also emerges as universally influential. This suggests that recent glucose levels are essential to identify glycemic events. The features based on the risk indices like LBGI (0-6h), and HBGI (0-6h) are significant, showcasing their significance in identifying low-glucose events and high-glucose events, respectively. Notably, some features (e.g., CHO and Bolus) show a bimodal or clustered pattern, underscoring their variable relevance across subpopulations. The asymmetry in impact direction suggests that the same feature may exert opposing effects depending on individual glucose dynamics, illustrating the benefit of personalized model partitioning. Interestingly, the Cluster feature showed substantial variability in attribution. While negative SHAP values predominated in some models, others assigned it a positive role—highlighting its heterogeneous influence and supporting its utility as a structural partitioning feature that captures behavioral or physiological subgroups

**Fig 6:**
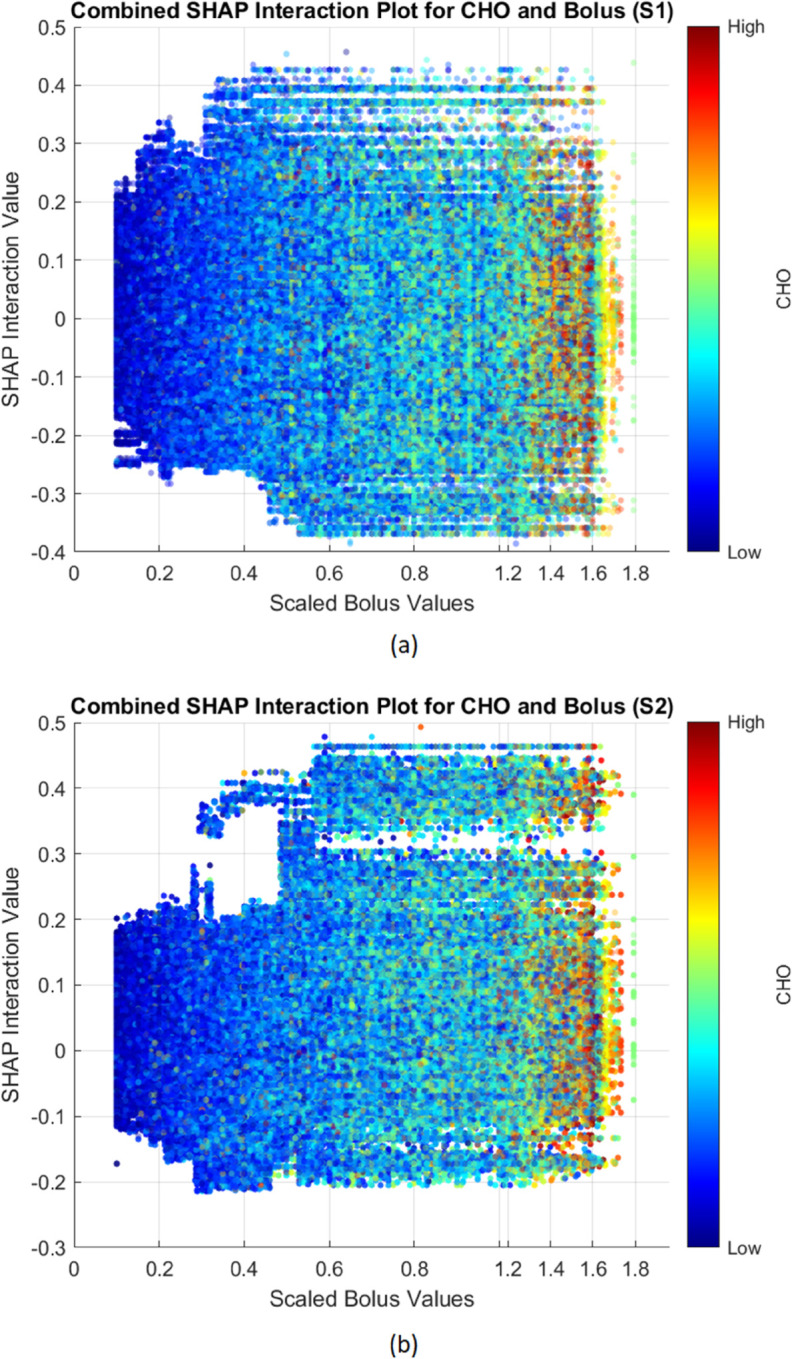
Combined SHAP interaction plots for CHO and Bolus across hypo (S1) and hyper (S2) models. The x-axis represents bolus values on a custom scale, while the y-axis represents the SHAP interaction values.

While the SHAP value plot was useful for identifying the individual importance of features, it may overlook the combined effects of features that interact with each other. In this study’s SHAP value analysis, CHO and Bolus did not emerge as highly influential when considered independently. However, by using SHAP interaction values, the joint influence of these two variables on model predictions is clearly revealed, highlighting their relevance in scenarios where their interaction plays a key role in glycemic event prediction. Fig 6 presents the SHAP interaction values for CHO and Bolus, with Fig 6a focused on hypoglycemia predictions and Fig 6b on hyperglycemia predictions.

In Fig 6a, high Bolus values combined with high CHO levels frequently lead to positive SHAP interaction values, suggesting that their combination increases the likelihood of hypoglycemic events. Conversely, when both Bolus and CHO are low, the interaction values tend to be negative, indicating a lower risk of hypoglycemia in such scenarios. At intermediate CHO levels, the interaction effects are more variable, likely reflecting the influence of additional contextual factors.

Fig 6b exhibits a similar trend in variability, though with some notable differences in interaction dynamics. For high CHO and Bolus values, SHAP interaction values are again predominantly positive, indicating increased risk of hyperglycemia. This may reflect situations where insulin doses are insufficient or mistimed relative to carbohydrate intake, leading to elevated glucose levels. The distribution of interaction values appears more concentrated than in the hypoglycemia case—particularly at intermediate CHO levels—suggesting a stronger dependence on the CHO–Bolus interaction for hyperglycemia predictions under these conditions.

**Fig 7:**
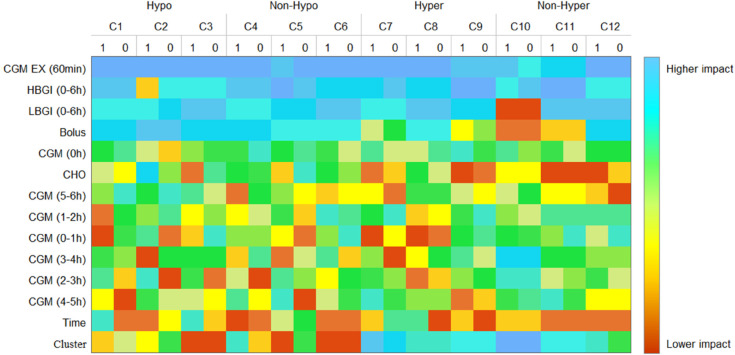
LIME-based feature importance for systems S1 and S2.

Finally, model-level interpretability was explored using LIME, which provides a local view of feature importance based on individual predictions. Fig 7 presents a heatmap of the average feature rankings across clusters for Systems S1 and S2. The heatmap reveals consistent patterns across clusters. Key features such as CGM EX (60min) and Bolus consistently rank highly, highlighting their critical role in predictive performance. Risk indices like LBGI (0-6h) and HBGI (0-6h) show variable importance, indicating a dependency on the model context or physiological differences captured by the data partitions. Recent glucose data (CGM 0-1h, 1-2h, 5-6h) ranks higher, while older intervals (CGM 4-5h, 2-3h) are less significant. Features like Time and Cluster generally rank lower, indicating limited influence. The patterns suggest distinct cluster groupings, emphasizing dependence on immediate glucose data and the universal importance of CGM EX (60min).

Overall, the results from LIME complement those obtained from SHAP, reinforcing that immediate glycemic trends and recent insulin–carbohydrate interactions are the dominant signals used by our models to anticipate glycemic excursions

### 3.3. Comparison with the state-of-the-art methods

Considering the importance of diabetes management, significant research has been conducted on postprandial hypoglycemia and hyperglycemia. While some studies have focused on predicting BG levels using time-series methods, others have concentrated on predicting specific hypoglycemic or hyperglycemic events during the postprandial period. The literature revealed that most research focuses primarily on predicting one event at a time—either hypoglycemia or hyperglycemia—with only a few studies addressing multi-class prediction involving both events simultaneously. Compared to existing state-of-the-art research on postprandial hypoglycemia and hyperglycemia prediction, our proposed system demonstrated promising results. To the best of our knowledge, none of the existing studies used the same dataset as used in this work. The comparison of the models is based on the performance metrics noted by the researchers. Table 4 summarizes the relevant state-of-the-art research on predicting postprandial hypoglycemia and hyperglycemia. From the table, although having a prediction horizon of 240 mins, the proposed methodology not only outperforms in terms of predictions and accuracy compared to other studies but also provides a rationale behind the predictions made by the model, making it more reliable and trustworthy.

**Table 4:** Summary of Studies on Postprandial Hypoglycemia and Hyperglycemia Prediction. (ANN: Artificial Neural Network, BG: Blood Glucose, DNN: Deep Neural Network, KNN: K-Nearest Neighbors, LightGBM: Light Gradient Boosting Machine, LR: Logistic Regression, LSTM: Long Short-Term Memory, NB: Naive Bayes, RF: Random Forest, SVC: Support Vector Classifier, SVM: Support Vector Machine.)

Author	Target Prediction	ML Model	Results	Explainability	Horizon
Proposed Method	Hypo & hyperglycemia	RF	Hypoglycemia: Accuracy: 86.50%, MCC: 0.47, Sensitivity: 72.77%, Specificity: 77.70%; Hyperglycemia: Accuracy: 85.60%, MCC: 0.73, Sensitivity: 82.04%, Specificity: 91.67%;	SHAP, LIME	240 min
Xin Xiong, et al. [37]	Glycemic response	LightGBM	Correlation (R): 0.63 (carbohydrate only: R=0.14; Insulin baseline: R=0.43)	SHAP	120 min
Giovanni Annuzzi, et al. [38]	BG levels	DNN	RMSE: 15 min - 20.5 mg/dL, 60 min - 32.1 mg/dL, 120 min - 41.3 mg/dL	SHAP	15, 60, 120 min
Ran Cui, et al. [39]	Hypo- & hyperglycemia	LSTM	Hypoglycemia: MCC: 0.48, Sensitivity: 48%, Specificity: 99%; Hyperglycemia: MCC: 0.61, Sensitivity: 66%, Specificity: 95%	No	30 min
Wonju Seo, et al. [40]	Hypoglycemia	RF, SVM, KNN, LR	Sensitivity: 89.6%, Specificity: 91.3%, AUC: 0.966, F1 Score: 0.543 (Best model: RF)	No	30 min
Silvia Oviedo, et al. [41]	Hypoglycemia	NB, AdaBoost, SVM, ANN	Sensitivity: 49%, Specificity: 74% (<70 mg/dL); Sensitivity: 51%, Specificity: 74% (<54 mg/dL)	No	240 min
Silvia Oviedo, et al. [42]	Hypoglycemia	SVC	Sensitivity: 71%, Specificity: 79% (Level 1 Hypoglycemia ≤70 mg/dL); Sensitivity: 77%, Specificity: 81% (Level 2 Hypoglycemia <54 mg/dL)	No	240 min

### 3.4. Assessment of the proposed approach in in silico environment

According to the proposed methodology, the bolus calculator was evaluated in an *in silico* environment, with results summarized in Table 5. The outcomes are categorized into overall and postprandial glycemic metrics, and reported as median values with interquartile ranges [IQR]. Statistical significance was assessed using the Wilcoxon matched-pairs signed-rank test, a non-parametric method suitable for paired comparisons.

**Table 5:** Glycemic outcomes before and after bolus adjustment.

Overall Glycemic Outcomes		
**Parameter**	**Before**	**After**
Median BG (mg/dL)${\;\;}^{*}$	119.4 [116.7–126.7]	127.0 [123.0–135.9]
Mean BG (mg/dL)${\;\;}^{*}$	122.2 [121.1–132.5]	133.1 [127.5–142.0]
Standard Deviation (mg/dL)${\;\;}^{*}$	43.3 [39.3–47.2]	43.8 [42.0–45.9]
Coefficient of Variation (%)${\;\;}^{*}$	36.0 [32.0–38.8]	33.3 [31.8–34.2]
% Time <54 mg/dL${\;\;}^{*}$	4.6 [2.2–7.3]	2.9 [1.2–4.6]
% Time 54–70 mg/dL${\;\;}^{*}$	4.2 [3.0–5.5]	2.7 [2.0–3.7]
% Time 70–180 mg/dL${\;\;}^{*}$	78.1 [75.0–84.3]	81.4 [77.9–83.0]
% Time >180 mg/dL${\;\;}^{*}$	6.8 [5.8–11.5]	11.2 [7.9–12.4]
% Time >250 mg/dL${\;\;}^{*}$	1.1 [0.6–2.4]	1.7 [0.7–3.4]
**Postprandial Glycemic Outcomes**		
**Parameter**	**Before**	**After**
Minimum BG (mg/dL)${\;\;}^{*}$	99.8 [96.5–106.8]	102.6 [99.7–108.2]
Maximum BG (mg/dL)${\;\;}^{*}$	166.7 [160.2–182.9]	177.5 [169.4–183.9]
Mean BG (mg/dL)${\;\;}^{*}$	139.3 [134.9–154.4]	150.1 [144.3–159.1]
Median BG (mg/dL)${\;\;}^{*}$	142.7 [136.4–155.3]	154.2 [147.7–163.2]
Initial BG (mg/dL)${\;\;}^{*}$	111.9 [105.8–117.3]	114.7 [108.8–119.9]
BG Excursion (mg/dL)${\;\;}^{*}$	10.8 [7.0–16.6]	15.1 [11.1–19.8]
Time to Peak (mins)${\;\;}^{*}$	102.5 [95–105]	107.5 [105–110]
% Time <54 mg/dL${\;\;}^{*}$	2.0 [1.3–3.4]	0.6 [0.3–2.1]
% Time 54–70 mg/dL${\;\;}^{*}$	2.3 [1.7–2.4]	1.3 [0.7–1.7]
% Time 70–180 mg/dL${\;\;}^{*}$	80.6 [71.5–83.3]	75.3 [71.7–80.5]
% Time >180 mg/dL${\;\;}^{*}$	13.4 [10.6–21.7]	18.2 [14.3–22.3]
% Time >250 mg/dL${\;\;}^{*}$	2.1 [1.2–4.8]	3.4 [1.3–6.6]

${\;\;}^{*}$p < 0.05

In the postprandial window, the adjusted bolus led to a reduction in the time spent below both 70 mg/dL and 54 mg/dL, indicating a measurable decrease in hypoglycemic exposure. Although a moderate increase was observed in hyperglycemic durations, the median percentages above 180 mg/dL (18.2) and 250 mg/dL (3.4) remained within acceptable limits established by current clinical consensus guidelines.

According to the proposed methodology, the bolus calculator was evaluated in the in silico environment, with results detailed in Table 5. The analysis categorizes outcomes into overall and post-prandial glycemic measurements, utilizing median [IQR] values. Statistical analysis used the Wilcoxon matched-pairs signed-rank test—a non-parametric approach tailored for paired data—to assess the intervention’s significance.

In the postprandial window, the adjusted bolus led to a reduction in the time spent below both 70 mg/dL and 54 mg/dL, indicating a measurable decrease in hypoglycemic exposure. Although a moderate increase was observed in hyperglycemic durations, the median percentages above 180 mg/dL (18.2) and 250 mg/dL (3.4) remained within acceptable limits established by current clinical consensus guidelines.

## 4. Discussion

The proposed two-tier clustering approach led to substantial improvements in the prediction of postprandial glycemic events over traditional machine learning models. Both systems, S1 and S2, significantly outperformed their respective baselines in terms of accuracy and correlation metrics, validating the benefits of stratified modeling. These gains are attributable to the clustering framework, which effectively segments patients into more homogeneous and profiled subgroups.

This segmentation effect is evident in ROC analysis: system S1 exhibited a considerable gain in average AUC compared to its baseline, while S2 achieved even higher discrimination. These improvements highlight the effectiveness of incorporating glycemic profiles into the training pipeline, particularly when working with heterogeneous patients.

Interpretability analysis using SHAP revealed important insights into feature relevance across different clusters. CGM features from immediate time windows (0h, 0–1h, and 1–2h) consistently appeared as key predictors of hypoglycemia. Additionally, the cluster label itself carried notable attribution scores, reinforcing the idea that stratified models better capture physiological variability. Risk indices such as the LBGI and HBGI further demonstrated condition-specific relevance, particularly in hyperglycemia predictions, where CHO contributions were also more pronounced.

Complementary analysis using LIME confirmed the dominant influence of recent CGM data on model predictions. Windows like 0–1h and 1–2h consistently ranked highest across clusters. However, due to its local scope, LIME proved less effective in summarizing global patterns or interaction effects, emphasizing the advantage of combining local and global interpretability tools.

Despite expectations, carbohydrate intake did not emerge as a dominant predictor in isolation. Its attenuated relevance may be explained by the strong direct signal captured through CGM, variability in insulin sensitivity, or inconsistencies in CHO logging—common in real-world data. This underlines the importance of capturing downstream physiological responses rather than relying solely on meal reports.

A notable advantage of the proposed methodology is its transparency and explainability. In contrast with many black-box approaches using deep learning or large ensembles, this framework enables interpretability both at the cluster level and per individual prediction. This aligns with growing demands for algorithmic transparency in clinical AI systems and increases the potential for real-world integration.

A practical consideration is the added complexity introduced by the cluster-specific modeling approach. Although training is performed offline, deploying multiple models in parallel could strain computation on resource-constrained platforms. Nonetheless, the use of efficient classifiers (e.g., random forests with shallow trees) supports low-latency inference. Future work may explore model compression techniques such as pruning or distillation to further optimize performance.

Building on these findings, a bolus adjustment protocol was evaluated in an *in silico* environment. Although simulations cannot fully replicate clinical complexity, extensive effort was made to capture critical aspects of variability observed in clinical practice, including timing, content, and physiological responses. This allowed for a realistic testbed to assess performance under diverse yet plausible conditions. The adjusted bolus strategy led to a meaningful reduction in postprandial hypoglycemia, with a modest and clinically acceptable increase in hyperglycemic exposure.

Importantly, this work provides a potential blueprint for integrating individualized glycemic profiles into bolus calculators and decision support tools. By tailoring predictions to patient subgroups and enhancing transparency, this approach could support more precise and trustworthy insulin recommendations in both clinical and self-managed contexts.

The study’s most significant contribution lies in demonstrating how advanced clustering techniques can substantially improve glycemic event prediction. By creating cluster-specific models, the approach addresses the inherent variability in individual glycemic responses, a critical challenge in personalized diabetes management. The explainable AI techniques not only validated the model’s performance but also provided transparent insights into the decision-making process, addressing a crucial barrier in clinical AI adoption.

Several limitations must be acknowledged. The study relied on retrospective data and in silico simulation, without prospective validation in real patients. Additionally, unmodeled factors such as stress, illness, or physical activity may limit generalizability. Future work should include multimodal data (e.g., from wearables), validation across diverse populations, and mechanisms for patient feedback and shared decision-making to increase usability and adherence.

## 5. Conclusion

This work presents an interpretable, cluster-based machine learning framework that enhances the prediction of postprandial glycemic events by incorporating glycemic profiling into model design. The combination of unsupervised patient stratification and feature-level explainability led to improved predictive performance, particularly for extended horizons, while offering transparency suitable for clinical settings. Simulation results confirmed the potential for model-informed bolus adjustment to reduce hypoglycemia without substantially increasing hyperglycemia. Overall, the approach represents a promising step toward adaptive and trustworthy decision support in diabetes management.

## Supporting information

S1 Dataset The datasets used in this study, including the REPLACE-BG dataset and the in silico simulated dataset, are provided in compressed format. These datasets were used to train, validate, and evaluate the proposed ML framework for the prediction of postprandial glycemic events and insulin dose optimization.
(ZIP)
